# Integrative network analysis identifies pivotal host genes and pathways for SARS-CoV-2 infection

**DOI:** 10.1016/j.gendis.2024.101206

**Published:** 2024-01-03

**Authors:** Gao Chen, Li Li, Ruiqi Wang, Bin Liu, Zhiwen Cao, Ning Zhao, Yong Tan, Xiaojuan He, Jing Zhao, Cheng Lu

**Affiliations:** a School of Life Science, Hubei University, Wuhan, Hubei 430062, China; b Institute of Basic Research in Clinical Medicine, China Academy of Chinese Medical Sciences, Beijing 100700, China

The coronavirus disease 2019 (COVID-19) is still a global threat today. SARS-CoV-2 is the etiologic agent of COVID-19, the culprit behind the current pandemic. Its life cycle is dependent on hijacking host-cell biological processes to facilitate entry, replication, assembly, and budding. The recognition that a suite of mammalian host proteins is required for SARS-CoV-2 infection and replication presents additional targeting strategies that may be less prone to deflections by the quickly mutating viral genome. In this study, we conducted an integrative network analysis to identify pivotal host genes and pathways for SARS-CoV-2 infection.

In this study, we systematically analyzed a large amount of RNA sequencing data from five molecular studies of SARS-CoV-2 infection, with multiplicity of infection (MOI) ranging from 0.01 to 4, and test time points ranging from 1 h to 48 h (see Table S1). Initially, we carried out a weighted gene co-expression network analysis, which yielded 31 modules (Fig. 1A; Fig. S1A–D). Subsequent correlation analysis results revealed that six modules were significantly associated with MOI, while no module showed a significant correlation with the time course (Fig. 1A), which was supported by the gene significance for MOI (Fig. S2A–F). Regression analysis results showed that no module was also associated with the experiment or the cell type (Fig. 1A), indicating that these gene modules were unaffected by the conditions of the experiments. Upon analysis of function enrichment, the genes in one of the six modules were enriched in the SARS-CoV-2 signaling pathway (Fig. S3B). Thereout, discovering these modules linked to SARS-CoV-2 infection was not an accidental event.

**Figure 1:**
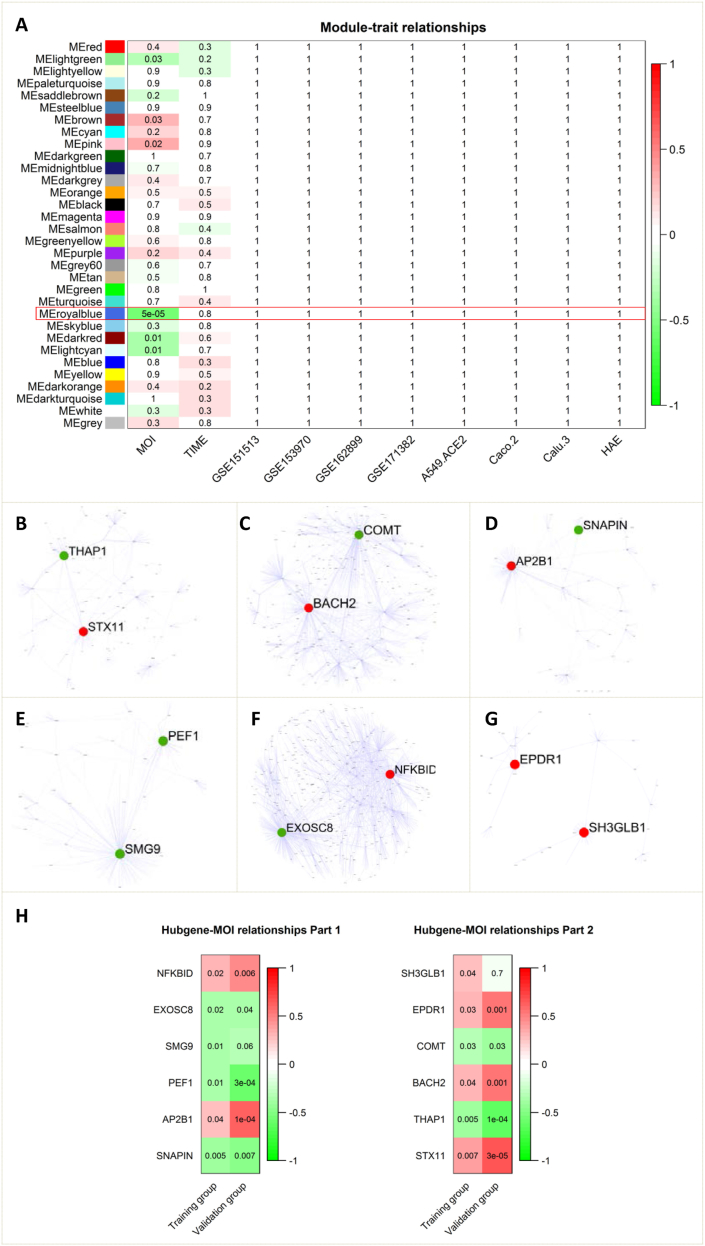
Integrative network analysis identifies pivotal genes. In a cell, there is a *P*-value and color-denoted coefficient. **(A)** The relationships between module eigengenes and traits. Each colored row in the *y*-axis represents a color-coded module. **(B**–**G)** The protein–protein interaction networks of the virus-related modules including, in turn, the royal blue module, pink module, light cyan module, dark red module, brown module, and light green module. The clearly labeled node was the top hub gene. **(H)** The relationships between top hub genes and traits. The validation group was from another independent experiment.

The royal blue module exhibited the highest correlation with MOI (Fig. 1A). The genes in this module were enriched in pathways related to the SARS-CoV-2 signaling, neuroinflammation, and neuron death (Fig. S3A, B). Evidence accumulated so far indicates that the pathophysiology of COVID-19 is characterized by systemic inflammation, hypoxia resulting from respiratory failure, and neuroinflammation (either due to viral neurotropism or in response to cytokine). Mice lacking THAP1 (THAP domain containing 1) exhibited neurological impairment.^1^ THAP1 was identified as a hub gene within the royal blue module upon analysis of a protein–protein interaction network (Fig. 1B). It had a negative correlation with MOI, which was confirmed in a dataset from an additional independent study (Fig. 1H). According to the centrality-lethality rule (in the protein–protein interaction network of a module, the higher the neighbor count is, the more important a node is), THAP1 is crucial in the neuroinflammation caused by SARS-CoV-2. Syntaxin 11 (STX11), another hub gene (Fig. 1B), was associated with hemophagocytic lymphohistiocytosis, a multisystem inflammatory disorder due to cytokine overproduction. IL-1 receptor antagonist is in many cases used to treat this sickness. IL-1 is initially released from the virus-infected cell and triggers ensuing inflammation. It was reported that a patient with hemophagocytic lymphohistiocytosis resulting from a homozygous frame-shift mutation of STX11 achieved remission with IL-1 receptor antagonist monotherapy, but subsequently developed an acute SARS-CoV-2 infection responsive to increased IL-1 receptor antagonist dosing.^2^ STX11 and MOI had a positive correlation in our study. Evidently, STX11 has a crucial role in the inflammation brought on by SARS-CoV-2.

The genes in the pink module (one of the six modules) were enriched in pathways related to MAPK signaling and JUN phosphorylation (Fig. S3C, D). BACH2 (BTB domain and CNC homolog 2) links MAPK/JUN and is a repressor of JUN-mediated transcriptional activation. Several studies demonstrated that vitamin *D* increased BACH2 to reduce IFN-γ and increase IL-10. These two cytokines are critical in resolving type 1 inflammation in the setting of severe COVID-19. BACH2, a hub gene in this module (Fig. 1C), had a positive correlation with MOI in this study (Fig. 1H), indicating that BACH2 negatively regulated inflammation caused by SARS-CoV-2. Another hub gene COMT (catechol-O-methyltransferase) (Fig. 1C) encoding catechol-O-methyltransferase catalyzes the transfer of a methyl group from S-adenosylmethionine to catecholamines, including the neurotransmitters dopamine, epinephrine, and norepinephrine. This enzyme was activated by the MAPK signaling pathway, relieving functional pain and neuroinflammation.^3^ Our study found that COMT was negatively correlated with MOI (Fig. 1H). COMT should keep down-regulation in the SARS-CoV-2 infection, resulting in functional pain and neuroinflammation.

SARS-CoV-2 enters through clathrin-mediated endocytosis, and its genome is trafficked to the early endosomes. The AP2B1 (beta-type subunit of the adaptor protein complex 2) coding protein is one of two large chain components of the adaptor protein complex 2, which serves to link clathrin to receptors in coated vesicles. AP2B1, a hub gene in the light cyan module (Fig. 1D), was positively correlated with MOI (Fig. 1H). Thus, AP2B1 expression should promote SARS-CoV-2 entry. On the other side, SARS-CoV-2 exploits the lysosomal exocytosis pathway for egress. The SNAPIN (SNAP-associated protein) encoding protein is a component of the BLOC-1, a complex that is required for the normal biogenesis of lysosome-related organelles. Its phosphorylation state influences exocytotic protein interactions and regulates exocytosis. This protein is phosphorylated by the MAPK cascade. In this study, the genes in the light cyan module were enriched in the MAPK cascade pathway (Fig. S3E), and SNAPIN (Fig. 1D) was negatively correlated with MOI (Fig. 1H). Obviously, SNAPIN is involved in the regulation of SARS-CoV-2 egress.

The SARS-CoV-2 assembly occurs in the endoplasmic reticulum-Golgi intermediate compartment. Endoplasmic reticulum-to-Golgi transport is the first step in the constitutive secretory pathway. PEF1 (penta-EF-hand domain containing 1) encoding protein ALG-2 (apoptosis-linked gene 2) is a negative regulator of endoplasmic reticulum-Golgi transport. Furthermore, ALG-2/peflin hetero-complexes bind to endoplasmic reticulum exit sites through the ALG-2 subunit to confer a low, buffered secretion rate.^4^ PEF1 as a hub gene in the dark red module (Fig. 1E) had a negative correlation with MOI (Fig. 1H) and the genes in this module were enriched in the endoplasmic reticulum to Golgi vesicle-mediated transport pathway (Fig. S3G). Therefore, PEF1 plays a key role in the SARS-CoV-2 assembly and secretion.

SARS-CoV-2 hijacks the exosomal pathway to exploit cellular replication mechanisms and further spread infection throughout the body. Exosomes are lipid bilayer-encapsulated microparticles (30–100 nm) released by various types of cells. The genes in the brown module were enriched in vesicle-mediated transport and lipid metabolic processes (Fig. S3I). Exosome component 8 (EXOSC8), a hub gene in this module (Fig. 1F), encodes a 3′–5′ exoribonuclease, part of the exosome complex. Meanwhile, exosomes allow the host to produce effective immunity by activating antiviral mechanisms.^5^ For example, exosomes were engulfed and led to the NF-κB signaling activation by hindering the NFKB inhibitor delta's (NFKBID),^5^ another hub gene (Fig. 1F). In this study, EXOSC8 and NFKBID were significantly correlated with MOI (Fig. 1H), suggesting that these two genes should be essential in the response to SARS-CoV-2 infection in terms of the exosome.

Ependymin-related 1 (EPDR1) overexpression increased the p53, p21, and Bcl-2 expression and promoted apoptosis. In this study, the genes in the light green module were enriched in the apoptotic process (Fig. S3K), and EPDR1 was positively correlated with MOI (Fig. 1H). As a hub gene in this module (Fig. 1G), EPDR1 is bound to play a pivotal role in the apoptosis caused by SARS-CoV-2.

In summary, by combining the weighted gene co-expression network approach, protein–protein interaction network analysis, and RNA sequencing data, this study uncovered fundamental patterns of molecular responses, intrinsic structures of gene co-regulation, and potential targets in SARS-CoV-2 infection. Our findings provided further insights into functional investigations to identify potential therapeutic targets against SARS-CoV-2 infection.

## Author contributions

Conceptualization, G.C. and C.L.; methodology, G.C.; software, G.C.; validation, L.L., R.W., and B.L.; formal analysis, Z.C.; data curation, N.Z.; writing-original draft preparation, G.C.; writing-review and editing, X.H.; visualization, Y.T.; supervision, C.L.; project administration, J.Z.; funding acquisition, C.L. All authors read and agreed to the published version of the manuscript.

## Conflict of interests

The authors declare no conflict of interests.

## Funding

This research was funded by the Innovation Team and Talents Cultivation Program Administration Traditional Chinese Medicine (No. ZYYCXTD-D-202005), Youth Qihuang Scholar of National Administration Traditional Chinese Medicine (2020), and Scientific and Technological Innovation Project of China Academy of Chinese Medical Sciences (No. CI2021B003).
