# Supplementary material for: Integrative network analysis identifies pivotal host genes and pathways for SARS-CoV-2 infection
Source: Genes Dis. 2024 Jan 3;12(1):101206. doi: 10.1016/j.gendis.2024.101206 (PMC11462195; doi:10.1016/j.gendis.2024.101206)
Supplement: Multimedia component 1 [file mmc1.docx]

**Materials and methods**

*Dataset and preprocessing*

In this study, five RNA sequencing files for SARS-CoV-2 infection were obtained from the Gene Expression Omnibus (GEO) repository (<https://www.ncbi.nlm.nih.gov/geo/>), containing GSE151513, GSE153970, GSE162899, GSE171382, and GSE184536. These files consisted of gene expression matrices and experiment designs. The main features of these datasets were shown in Table S1 (Table S2).

All of the raw data of GSE151513, GSE153970, GSE162899, and GSE171382 were merged and normalized into the training group through a unified ENCODE RNA-seq processing pipeline, followed by removing the batch effect using the Limma package of R ^[1](#_ENREF_1)^. A multi-dimensional scaling plot was used to estimate whether the batch effect was removed (Fig. S4). The validation group was the dataset from GSE184536.

*Identification of significant modules using the Weighted Gene Co-expression Network Analysis*

The WGCNA package ^[2](#_ENREF_2)^ was used to determine key genes significantly associated with SARS-CoV-2 infection in the training and validation groups. The best soft threshold power was set to identify the module-trait relationship, module membership (MM), and gene significance (GS). In brief, a weighted adjacency matrix was first constructed based on the selected soft threshold power of 6 (Fig. S5). Subsequently, the connectivity per gene was deduced by calculating connection strengths with other genes. To resolve computational challenges caused by constructing and analyzing networks with such large numbers of nodes, function blockwiseModules in the WGCNA package was used to split into two blocks for hierarchical clustering (Fig. S1A, B). The hierarchical clustering trees were constructed following a dynamic hybrid cut. Thirty-one modules were identified when the DissThres was set as 0.25 after merging dynamic modules, as shown in the clustering dendrograms (Fig. S1C). The gene expression profile of each module was summarized by the module eigengene, which refers to the PC1 of the expression level in the genes from a module. The Pearson correlation analysis was conducted to examine the relationship among gene modules. The eigengene dendrogram and heatmap were used to quantify module similarity by eigengene correlation (Fig. S1C, D). The GS and MM values were also generated. GS stands for the level of correlation between gene expression and designated features. MM stands for the correlation of the module eigengene and the gene expression profile.

The study traits were regressed on the module eigengene in the Limma R package ^[3](#_ENREF_3)^. The correlation between module eigengenes and time course or MOI was calculated for key module selection based on Pearson correlation coefficient (PCC). The function corPvalueStudent was used to calculate the Student asymptotic P-value. With all statistical methods, p < 0.05 was considered statistically significant.

*Function enrichment analysis*

An online tool (https://david.ncifcrf.gov/) was employed to perform a Function Enrichment Analysis of the genes in the significant modules ^[4](#_ENREF_4)^. The Gene Ontology (GO) and Kyoto Encyclopedia of WikiPathways analysis were conducted for the exploration of the involved biological functions and pathways of the above genes. The items ranked within the top 10 according to p-value were selected and visualization was conducted using the barplot function in R software.

*Protein-protein interaction network analysis*

The Search Tool for the Retrieval of Interacting Genes (The Human Reference Interactome and Literature Benchmark, HuRI, http://interactome-atlas.org) ^[5](#_ENREF_5)^ was used to identify interactions between the products of the genes in the modules. The PPI network was constructed using HuRI by adopting the default threshold and visualized using Cytoscape 3.8.2. The connection degree (number) of each node was calculated using the cytoHubba plugin ^[6](#_ENREF_6)^ within Cytoscape. In each PPI network, we selected the top 2 hub genes ranked with connection degrees in the genes that their expression was correlated with MOI in the training group (Fig. 1), which guaranteed that there was a representative gene in each module at least.

*The correlation between expression levels of hub genes and traits*

The Pearson correlation analysis was conducted to examine the relationship between expression levels of hub genes and time course or MOI in the training and validation groups, respectively. P-value <0.05 was considered statistically significant.

**Reference**

1. Zhou W, Koudijs KKM, Bohringer S. Influence of batch effect correction methods on drug induced differential gene expression profiles. *BMC Bioinformatics.* 2019;20(1):437.doi:10.1186/s12859-019-3028-6

2. Langfelder P, Horvath S. WGCNA: an R package for weighted correlation network analysis. *BMC Bioinformatics.* 2008;9:559.doi:10.1186/1471-2105-9-559

3. Yuan B, Liu W, Huo M, et al. The hub ten gene-based risk score system using RNA m(6)A methylation regulator features and tumor immune microenvironment in breast cancer. *Breast Cancer.* 2022;29(4):645-658.doi:10.1007/s12282-022-01341-5

4. Sherman BT, Hao M, Qiu J, et al. DAVID: a web server for functional enrichment analysis and functional annotation of gene lists (2021 update). *Nucleic Acids Res.* 2022.doi:10.1093/nar/gkac194

5. Luck K, Kim DK, Lambourne L, et al. A reference map of the human binary protein interactome. *Nature.* 2020;580(7803):402-408.doi:10.1038/s41586-020-2188-x

6. Chin CH, Chen SH, Wu HH, Ho CW, Ko MT, Lin CY. cytoHubba: identifying hub objects and sub-networks from complex interactome. *BMC Syst Biol.* 2014;8 Suppl 4:S11.doi:10.1186/1752-0509-8-S4-S11
