# Supplementary material for: Integrative network analysis identifies pivotal host genes and pathways for SARS-CoV-2 infection
Source: Genes Dis. 2024 Jan 3;12(1):101206. doi: 10.1016/j.gendis.2024.101206 (PMC11462195; doi:10.1016/j.gendis.2024.101206)
Supplement: Multimedia component 2 [file mmc2.docx]

**Table S1**  The main features of 5 selected datasets in this analysis.

| GEO datasets | Sample Size | Cell type | MOI | Time point (hours) |
| --- | --- | --- | --- | --- |
| GSE151513 | 33 | Calu-3 | 0; 2 | 1; 2; 3; 6; 12 |
| GSE153970 | 6 | HAE | 0; 0.5 | 48 |
| GSE162899 | 8 | Caco-2 | 0; 4 | 3; 6 |
| GSE171382 | 3 | A549-ACE2 | 0; 0.1 | 24 |
| GSE184536 | 34 | A549-ACE2 | 0; 2 | 2; 6; 12; 24 |

**Table S2** The selected samples from 5 GEO datasets in this analysis

| Datasets | Size | The selected samples |
| --- | --- | --- |
| GSE151513 | 33 | GSM4579947- GSM4579963; GSM4579965- GSM4579982 |
| GSE153970 | 6 | GSM4661080- GSM4661085 |
| GSE162899 | 8 | GSM4964871- GSM4964867 |
| GSE171382 | 3 | GSM5224710- GSM5224712 |
| GSE184536 | 34 | GSM5592073- GSM5592092; GSM5592094- GSM5592108 |

| 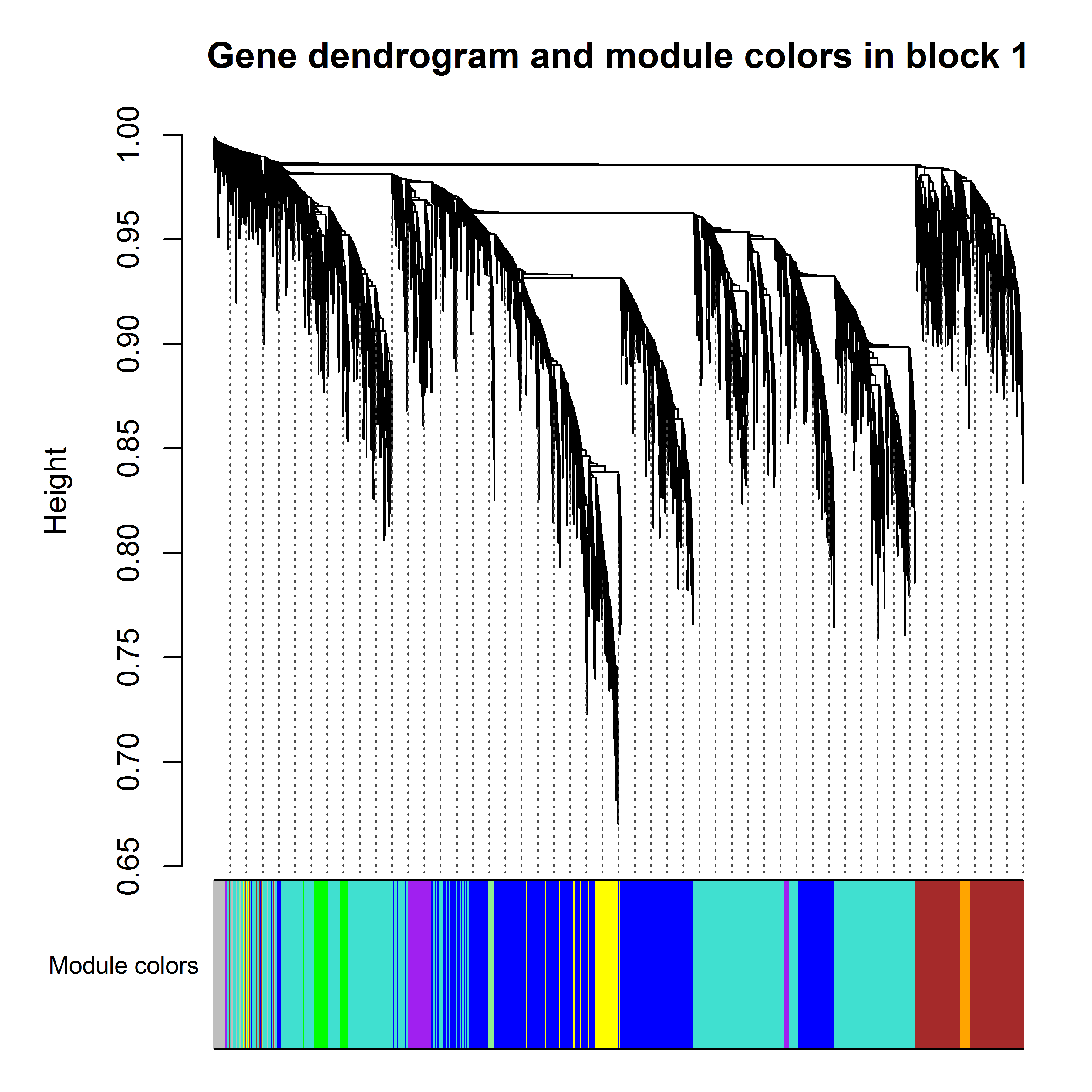  **C**  **A** | 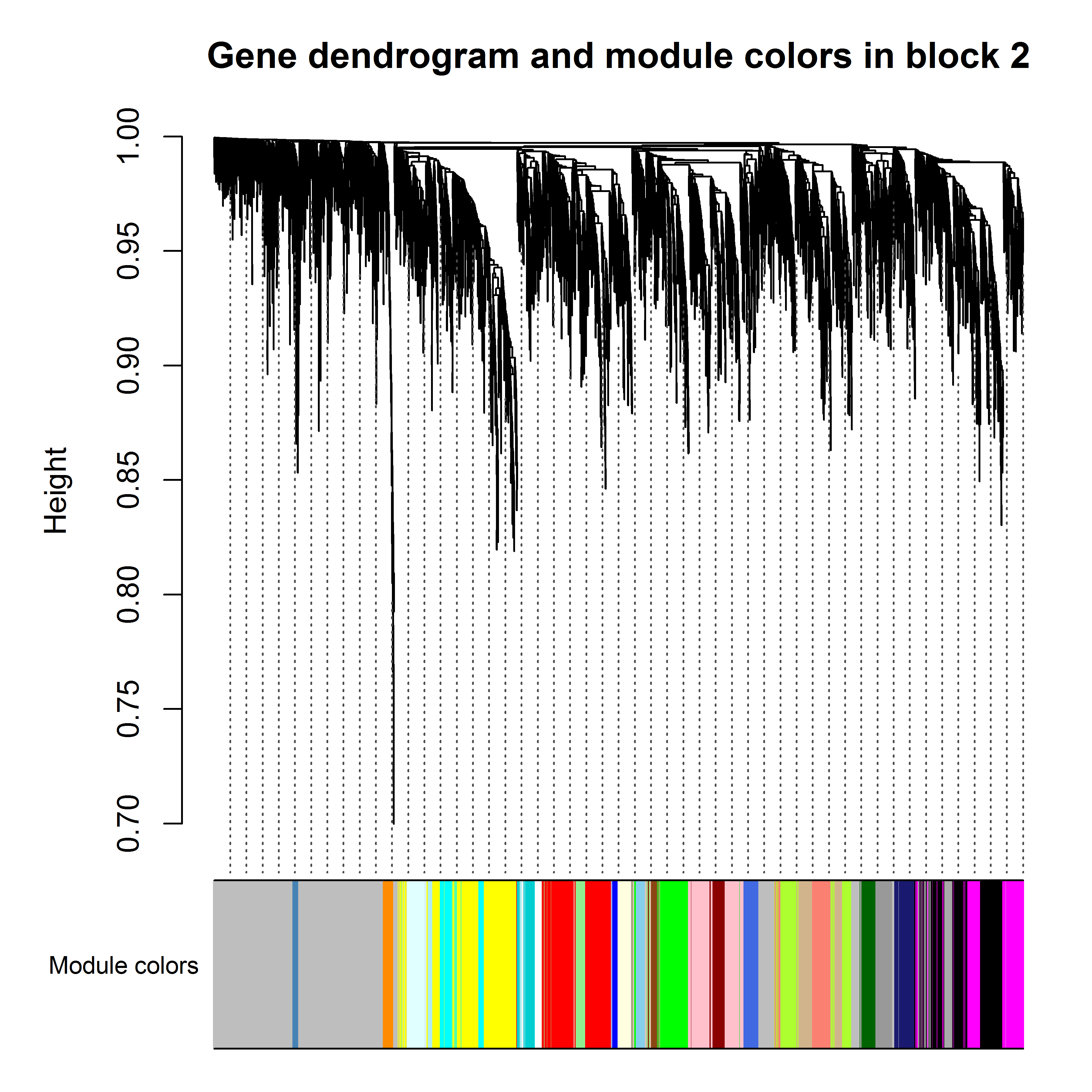  **D**  **B** |
| --- | --- |
| 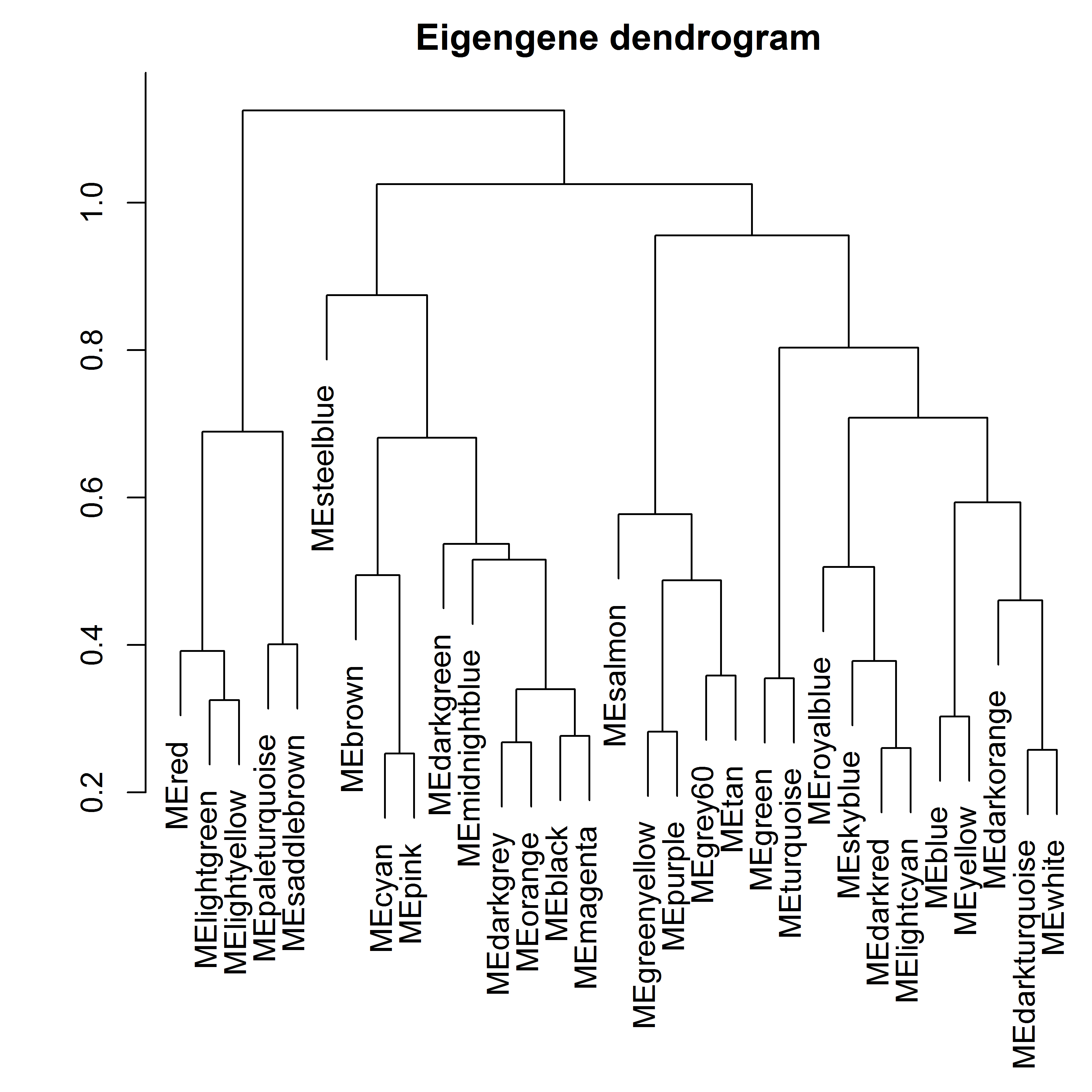 | 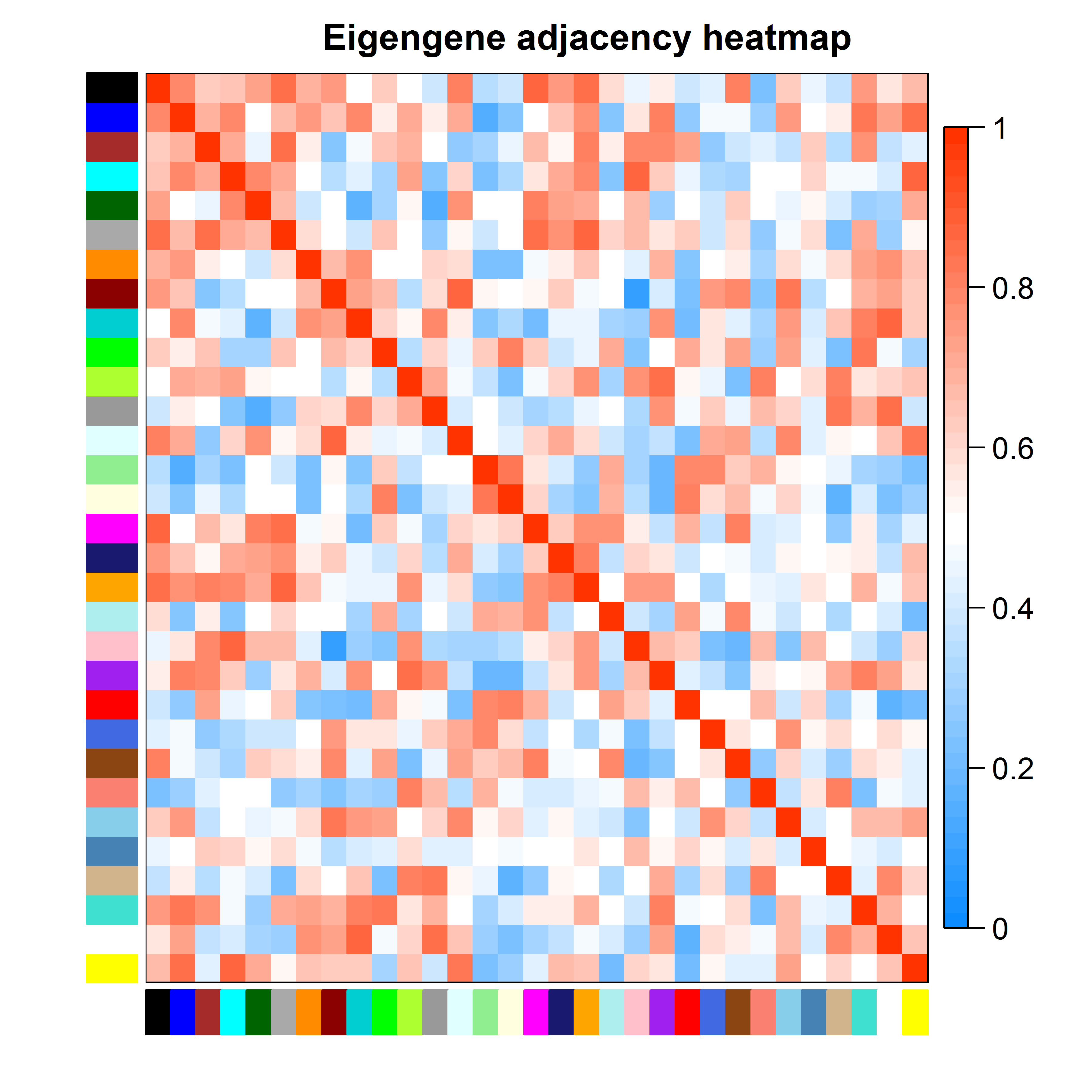 |

**Figure S1** Module detection and network heatmap plot construction using WGCNA in the training group. Each colored row represents a color-coded module that contains a group of highly connected genes. (A, B) The dendrogram obtained by hierarchical clustering of genes based on their topological overlap is shown at the top. (C) Dendrogram of module eigengenes obtained from WGCNA on the correlation. (D) Module eigengene adjacency heatmap (each colored row in the x- or y-axis represents a color-coded module).

| 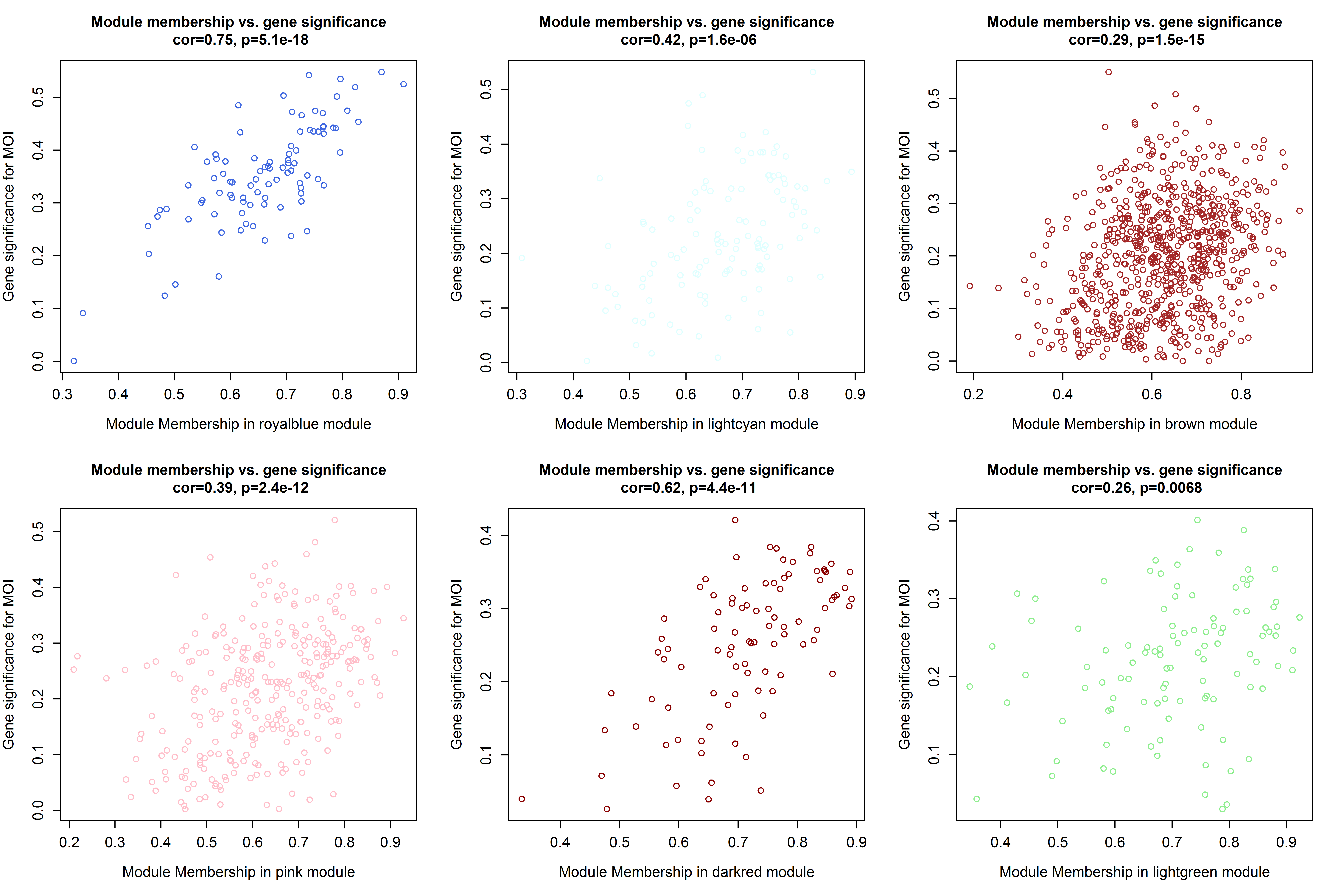  **F**  **E**  **C**  **B**  **D**  **A** |
| --- |

**Figure S2** The scatterplot of Gene Significance (GS) for weight vs. Module Membership (MM) in the 6 virus-related modules.

**B**

**A**

| 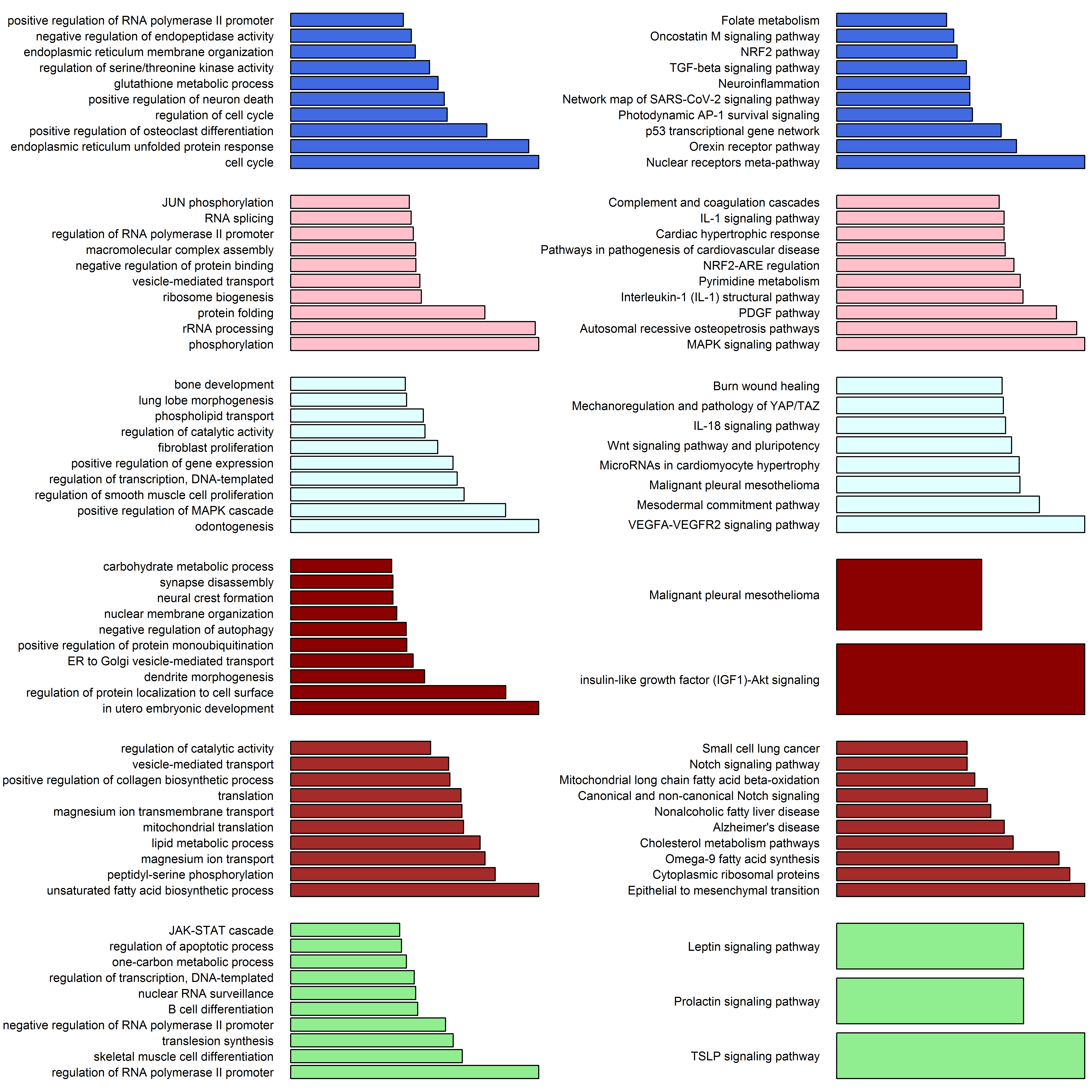  **E**  **F**  **K**  **L**  **J**  **I**  **H**  **G**  **D**  **C** |
| --- |

**Figure S3** GO and WikiPathways enrichment analysis of module genes in the training group. The length of bars displays –log10(p-value). (A) Royalblue module: GO. (B) Royalblue module: WikiPathways. (C) Pink module: GO. (D) Pink module: WikiPathways. (E) Lightcyan module: GO. (F) Lightcyan module: WikiPathways. (G) Darkred module: GO. (H) Darkred module: WikiPathways. (I) Brown module: GO. (J) Brown module: WikiPathways. (K) Lightgreen module: GO. (L) Lightgreen module: WikiPathways.


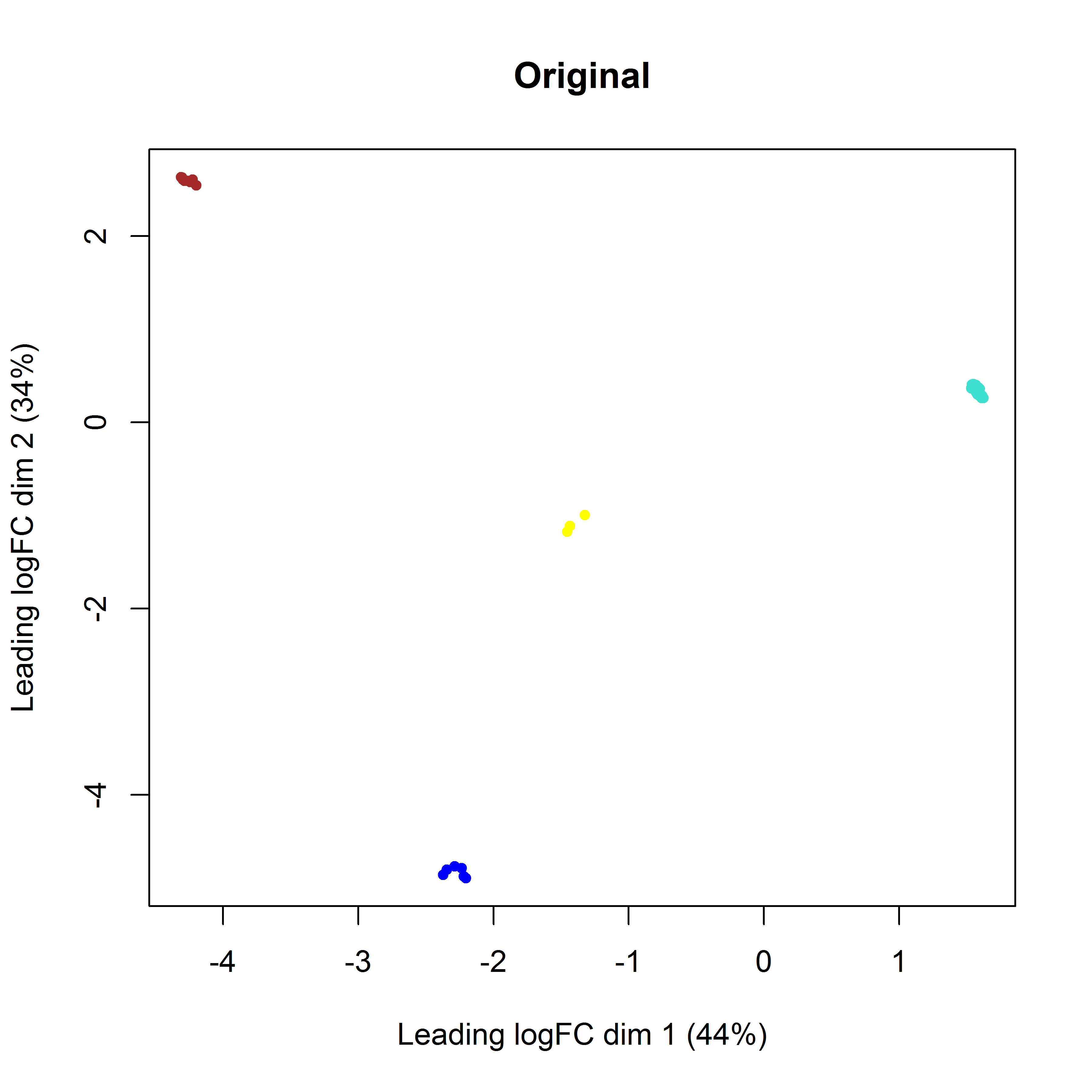

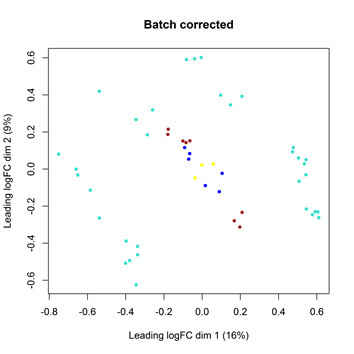


**A**

**B**

**Figure S4**  A multi-dimensional scaling plot. (A) Original data. (B) Batch-corrected data

**Figure S5** Determination of soft-thresholding power
